# Podophyllic Aldehyde, a Podophyllotoxin Derivate, Elicits Different Cell Cycle Profiles Depending on the Tumor Cell Line: A Systematic Proteomic Analysis

**DOI:** 10.3390/ijms25094631

**Published:** 2024-04-24

**Authors:** Ángela-Patricia Hernández, Lorea Chaparro-González, Olga Garzo-Sánchez, Carlota Arias-Hidalgo, Pablo Juanes-Velasco, Pablo A. García, Mª Ángeles Castro, Manuel Fuentes

**Affiliations:** 1 Department of Medicine and General Cytometry Service-Nucleus, CIBERONC CB16/12/00400, Cancer Research Centre (IBMCC/CSIC/USAL/IBSAL), IBSAL, University of Salamanca-CSIC, Campus Miguel de Unamuno s/n, 37007 Salamanca, Spain; loreachg@gmail.com (L.C.-G.); ogarzosanchez@gmail.com (O.G.-S.); carlotaariashidalgo@usal.es (C.A.-H.); pablojuanesvelasco@usal.es (P.J.-V.); mfuentes@usal.es (M.F.); 2 Department of Pharmaceutical Sciences, Laboratory of Medicinal Chemistry, Faculty of Pharmacy, University of Salamanca, Campus Miguel de Unamuno s/n, 37007 Salamanca, Spain; pabloagg@usal.es (P.A.G.); macg@usal.es (M.Á.C.); 3 Proteomics Unit, Cancer Research Centre (IBMCC/CSIC/USAL/IBSAL), 37007 Salamanca, Spain

**Keywords:** natural products, podophyllic aldehyde, podophyllotoxin, etoposide, functional proteomics, antibody microarray, cell cycle, high-throughput screening

## Abstract

When new antitumor therapy drugs are discovered, it is essential to address new target molecules from the point of view of chemical structure and to carry out efficient and systematic evaluation. In the case of natural products and derived compounds, it is of special importance to investigate chemomodulation to further explore antitumoral pharmacological activities. In this work, the compound podophyllic aldehyde, a cyclolignan derived from the chemomodulation of the natural product podophyllotoxin, has been evaluated for its viability, influence on the cell cycle, and effects on intracellular signaling. We used functional proteomics characterization for the evaluation. Compared with the FDA-approved drug etoposide (another podophyllotoxin derivative), we found interesting results regarding the cytotoxicity of podophyllic aldehyde. In addition, we were able to observe the effect of mitotic arrest in the treated cells. The use of podophyllic aldehyde resulted in increased cytotoxicity in solid tumor cell lines, compared to etoposide, and blocked the cycle more successfully than etoposide. High-throughput analysis of the deregulated proteins revealed a selective antimitotic mechanism of action of podophyllic aldehyde in the HT-29 cell line, in contrast with other solid and hematological tumor lines. Also, the apoptotic profile of podophyllic aldehyde was deciphered. The cell death mechanism is activated independently of the cell cycle profile. The results of these targeted analyses have also shown a significant response to the signaling of kinases, key proteins involved in signaling cascades for cell proliferation or metastasis. Thanks to this comprehensive analysis of podophyllic aldehyde, remarkable cytotoxic, antimitotic, and other antitumoral features have been discovered that will repurpose this compound for further chemical transformations and antitumoral analysis.

## 1. Introduction

Drug therapy, along with surgery, radiotherapy, and biological therapy, is still the leading approach for cancer treatment. Despite the current widespread knowledge of chemotherapy, the discovery of new targets for cancer treatment remains a goal of drug discovery [1]. The complexity of this disease requires targeted and effective approaches in terms of both chemical design and biological features [2,3].

Among the existing strategies for rational drug design, chemically modulating the activity of available drugs is a very successful approach in the field of medicinal chemistry [4,5]. Specifically, in natural product-based structures for cancer therapy, a great number of molecules have been identified as “hit compounds” and employed as a starting point for further chemical modifications; this number is still increasing [6]. 

**Figure 1:**
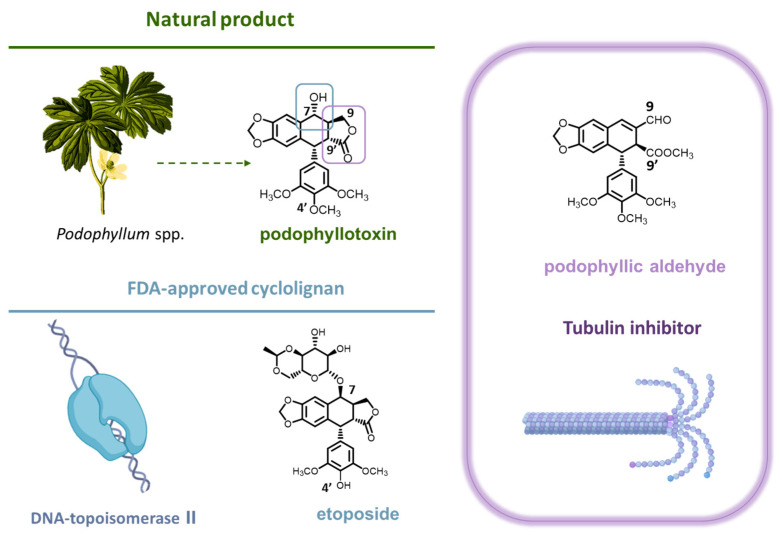
Structures of podophyllotoxin (indicating their most relevant positions with numbers) and the two semisynthetic derivatives etoposide and podophyllic aldehyde. The figure also represents the natural source of podophyllotoxin (*Podophyllum* spp.) and the corresponding targeted proteins for the compounds studied in this work: DNA topoisomerase II for etoposide and microtubules for podophyllic aldehyde.

An example of profitable results by chemical modification is the case of the natural product podophyllotoxin (Figure 1). Based on traditional medicine, podophyllotoxin, a cyclolignan obtained from species of the genus *Podophyllum*, has been used both for its intrinsic cytotoxic effect and, subsequently, for its chemomodulation activity [7]. As a result of its features, this natural compound is used in the treatment of venereal warts and has led, by chemical transformations, to the FDA-approved compound etoposide [8]. The structural modification of etoposide with respect to the podophyllotoxin skeleton is marked by epimerization and the inclusion of a bulky substituent at the C-7 position (Figure 1). This modified structure favors the biopharmaceutical and pharmacological profile with respect to the natural product (the side effects of systemic administration of podophyllotoxin are avoided) and led to the inclusion of this compound in chemotherapeutic protocols. Furthermore, from the point of view of the structure–activity relationship of podophyllotoxin-based cyclolignans, it offered a new perspective. Etoposide is a DNA topoisomerase II inhibitor [9,10], as contrasted with podophyllotoxin, which is a potent antimitotic that inhibits tubulin assembly [11]. Multiple chemomodulation strategies have been performed on podophyllotoxin in an attempt to mimic the structural modifications of etoposide to obtain new DNA topoisomerase II inhibitors [12,13]. However, modulating podophyllotoxin in an attempt to promote its potent antimitotic effect is also of particular relevance.

A key chemical feature of the natural product podophyllotoxin is the presence of a γ-lactone forming the aryltretraline skeleton [8]. This aggrupation was presumed to be essential for interaction with β-tubulin, leading to inhibition of microtubule polymerization. However, previous studies involving lactone cleavage and functionalization of the chemical groups have yielded compounds that maintain antimitotic and cytotoxic potency [14,15]. Among them, a compound with a methyl ester in C-9’ and presenting an α,β-unsaturated aldehyde grouping at the C-9 position was highlighted for its selectivity and far-reaching podophyllotoxin activity (IC_50_ at the nanomolar level). Several examples of chemomodulation of this compound, named podophyllic aldehyde (Figure 1), support the relevance of this small molecule as a promising lead compound for the development of new therapies based on lactone-lacking cyclolignans [16]. However, despite the background of etoposide and other podophyllotoxin-derived cyclolignans, the effect at the cell signaling level triggered by this chemomodulation on its targets remains to be elucidated. More assessments are required for an in-depth understanding of its various activities on the cyclolignan skeleton. 

**Figure 2:**
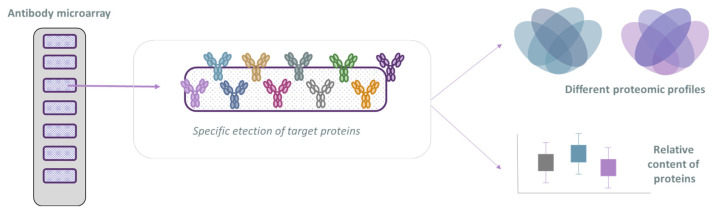
Representation of an antibody microarray and subsequent analysis.

Unraveling the complex challenge of understanding a drug’s mechanism of action in cancer therapy requires multiple strategies to decipher the intracellular signaling of newly emerging entities. Functional proteomics is one of the *omics* that is of particular relevance in drug discovery [17,18]. Knowledge of the relative protein levels is essential in order to map intracellular signaling and to respond to the particularities of each drug. High-throughput screening of proteins has made it possible to decode interactions of drugs and the microenvironment, decipher drug resistance mechanisms, and enable the building of proteomic profiles that can be correlated with the targeted pathologies [19,20,21]. For this purpose, antibody microarrays have been shown to serve as a useful tool in drug design and biomarker discovery [22,23]. These types of nanodevices provide a great amount of information from a small sample size. They are also affordable and yield reproducible and comparable results. Furthermore, they provide rapid detection of the targeted protein by the specific antibody bound onto the surface (Figure 2) [24]. 

To decipher the specific proteomic profile of podophyllic aldehyde (PA) as a lead compound, here we propose a comprehensive screening of this compound in several tumor cell lines, evaluating first its cytotoxicity and influence in cell cycle and then selecting the most highlighted conditions to further explore new proteomics insights. Aiming to provide a broader view of the activity of the cyclolignan skeleton, and given its importance in clinical practice, the activity of PA is correlated with the drug etoposide (ETO). With this study, particularities and similarities will be systematically explored, considering both pharmacological entities that share structural requirements but present different mechanisms of action. 

## 2. Results

### 2.1. Cytotoxic Analysis and Cell Cycle Evaluation

The first operation in the antitumoral profile analysis was the evaluation of the cytotoxic effect of ETO and PA in different tumor cell lines. Our study focused on the search for the antitumor mechanisms of PA compared to ETO. In this sense, taking into account the safety and proven efficacy of ETO in chemotherapy and against different types of tumors [25,26], different and diverse tumor lines have been included in order to explore the derived mechanisms of action. PA is a compound previously studied by our group, including various tumor lines (A549, HT-29, and MCF-7) [14,16]. In other studies, we tested PA in non-tumor cell lines, and the absence of toxicity was confirmed [27].

In order to broaden perspectives and find new selectivity trends, other lines of the same tumor type have been included: H460, Caco-2, and MDA-MB-231. In addition, hematological lines that had not previously been studied with PA and that are one of the applications of ETO have been added. Thus, cytotoxicity was explored in four different types of neoplasia: lung cancer (A549 and H460), colorectal cancer (HT-29 and Caco-2), breast cancer (MCF-7 and MDA-MB-231) and hematologic neoplasia (Jurkat for T cells and Ramos for B cells). In our previous experience with PA, we have observed that there is a great difference in the behavior of the compound when antitumoral effect is explored at different times of incubation [16]. Based on previous reports [14,15,16], we have chosen 72 h of incubation as the endpoint that ensures the correlation of cytotoxicity with other parameters studied [16]. Also, PA shows great chemical stability at long incubation times, since the potential oxidation of this product is nearly inactive [28]. 

In terms of IC_50_ values (Table 1), overall, it appears that PA is more cytotoxic than ETO in solid tumors, while the opposite is observed in the analyzed immune cells. In terms of the tumors studied, we were able to observe that there is a certain selectivity between studied tumor cell lines of the same pathology, with one of the two compounds studied having a greater cytotoxic effect than the other in the most cases. Also, we observed a decrease in IC_50_ of nearly 50-fold, or even more, in PA, compared to ETO in HT-29, which showed a 10-fold decrease in A549, H460, Caco-2, and MCF-7. On the other hand, the hematological lines were the least sensitive, with none of the tested conditions reaching an IC_50_ in the sub-micromolar range.

**Figure 3:**
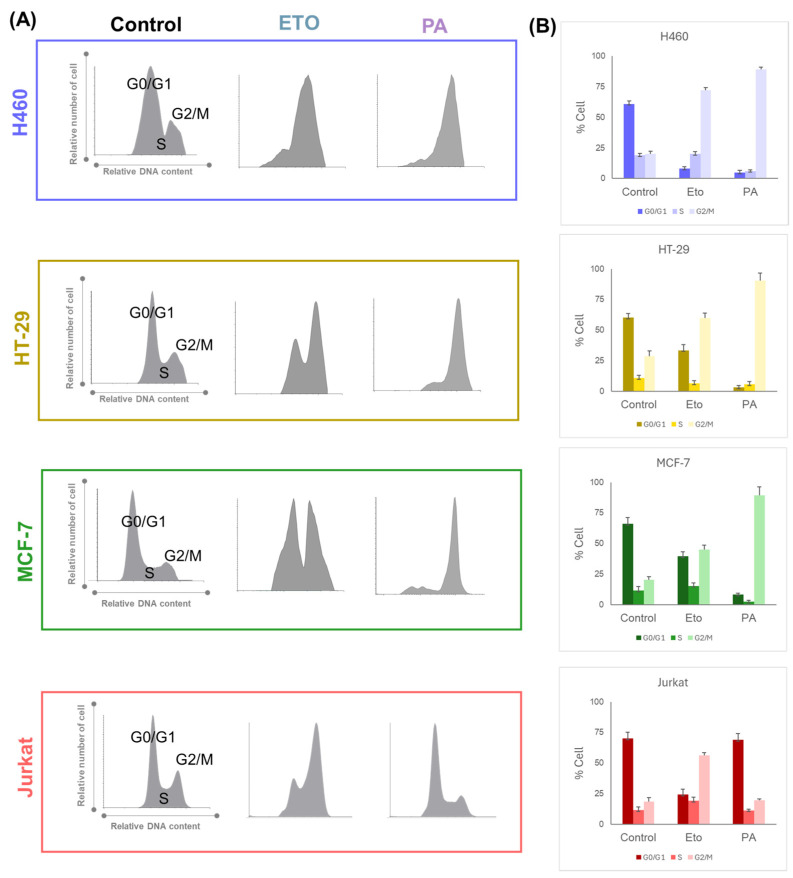
(**A**) Cell cycle profile of etoposide (ETO) and podophyllic aldehyde (PA) in selected tumor cell lines H460, HT-29, MCF-7, and Jurkat. (**B**) Cell cycle analysis indicating the % of cells in G0/G1; S and G2/M is detailed for each condition (results from two independent experiments expressed with SD).

Cell cycle analysis was conducted with the most sensitive cell line in the cytotoxic assay for PA within each type of tumor (lowest IC_50_ for the cell lines tested). Following our experience in previous studies [14,15,16], we conducted cell cycle assays at shorter times of incubation (24 h). PA is able to successfully show cell cycle arrest at 24 h, and this blockade correlates with the cytotoxicity observed at longer incubation times. Taking into account these prior results, ETO and PA were evaluated at 1 µM and cell cycle phases and relative number of cells in G0/G1, S, and G2/M were quantified and depicted in Figure 3. 

As can be observed in the cell cycle diagrams, PA totally arrested the cell cycle in the G2/M phase in solid tumor cell lines H460, HT-29, and MCF-7; meanwhile, in the Jurkat cell line, the cell cycle profile remained unaltered. ETO has a more heterogeneous profile depending on the cell line. In terms of the % of cells in the G2/M phase, the MCF-7 cell line presented a partial block where there was a similar number of cells in the G0/G1 phase and in the G2/M phase. For its part, in HT-29 and the Jurkat cell line, ETO increased the G2/M cell number with respect to G0/G1. In the case of the lung tumor line H460, a complete cycle blockade was observed with ETO treatment. The identification of these different profiles of cytotoxicity and cell cycle arrest encourage us to explore these two aspects further in a more detailed study of the intracellular proteomic profile.

### 2.2. Functional Proteomic Profiling of Etoposide and Podophyll Aldehyde for Cell Death Mechanisms

**Figure 4:**
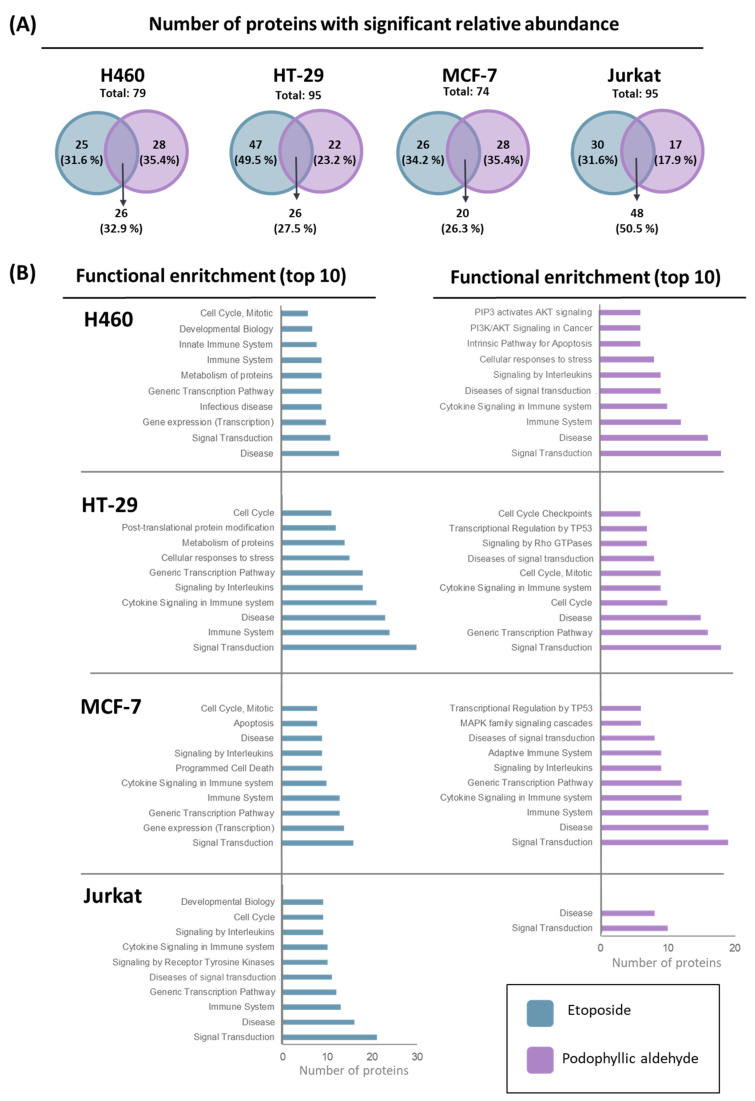
Proteomics overview for etoposide (blue) and podophyllic aldehyde (purple). (**A**) Venn diagrams represent the number of deregulated proteins (% in brackets) produced with respect to the control for both compounds. The diagram shows the number of unique proteins for etoposide and podophyllic aldehyde and the number of shared proteins. (**B**) Enrichment analysis: functions related to deregulated unique proteins for both compounds, showing functions with the highest number of proteins (top 10) and the number of proteins for each function.

ETO and PA showed a differential proteomic profiling in tumor cell lines H460, HT-29, MCF-7, and Jurkat under protein microarray-based analysis (Figure 4). 

For this purpose, tumor cells were treated and cell lysate was incubated in a protein microarray customized for the study of intracellular signaling pathways [29]. After incubation, a preliminary formal analysis was performed, obtaining the total number of proteins that were significantly deregulated with respect to the control conditions (cells without treatment). This study was primarily focused on determining the specific profile for each of the compounds tested. Therefore, significant proteins between the two profiles were compared side by side, and specific selected proteins for each profile were depicted in Venn diagrams (Supplementary File S1 contains the detailed list of proteins).

At first glance, interesting differences between the cell lines and the compounds can be observed. In two cell lines, H460 and MCF-7, there is a similar number of deregulated proteins for both compounds. However, in the cell lines HT-29 and Jurkat, it seems that a higher alteration at the protein level is observed in the ETO profile in comparison with PA. Also, there is a correlation in the total number of altered proteins observed for the compounds in the cell lines; this number was higher in the HT-29 and Jurkat cell line (95 deregulated proteins in both) compared with H460 and MCF-7 (79 and 74, respectively). Another observation regarding the number of downregulated proteins concerns the number of proteins shared by both compound profiles. In solid tumor cell lines, ETO and PA shared a similar number of proteins, but in the Jurkat cell line, the number was increased. In this case, the number of proteins was doubled compared with those of solid tumors.

To decipher a more detailed profile of the proteins deregulated by each of the compounds, a functional enrichment was carried out with those particular proteins deregulated by ETO and PA. In Figure 4B, the biological functions that presented a higher number of proteins for each of them is shown. In this case, different implications can be observed for the compounds. As expected, protein microarray content (Supplementary File S2) can lead one to decipher alterations in common cellular functions (such as disease, signal transduction, etc.); however, it was possible to establish and identify trends by analyzing in more detail the top 10 functions obtained in proteomic enrichment.

Comparing ETO and PA in the H460 line, PA presents a specific profile based on apoptosis, cell stress, and signaling through the AKT pathway, while ETO only presents cell cycle function as a specific pattern of cell death. In the HT-29 cell line, cycle inhibition is also repeated for ETO. In the case of PA, up to three functions have been found within the top ten functions related to the cell cycle, which may suggest a great weight for this type of signaling for this compound in this cell line.

Concerning the analysis of MCF-7, the ETO profile again shows the cell cycle function, as well as two others related to the process of cell death by apoptosis (apoptosis and programmed cell death). However, no specific cell death functions are observed in the PA profiles, despite the cytotoxicity and cell cycle results obtained. This is highlighted, in this case, in the way that immune-related processes are represented in the top 10 functions. Finally, in the Jurkat cell line, ETO also exhibits cell cycle function, as do the previous lines. In this case, PA presented a very limited number of specific proteins that did not result in particular functions in the enrichment conducted, apart from the general ones. As can be seen in all cell lines, cell cycle-related functions were prominent in the profile of ETO, which is related to its usual mechanism as a DNA topoisomerase II inhibitor. This compound showed homogenous signaling transversely in the cell lines studied. However, in the case of PA, it seems that signaling is more heterogeneous among cell lines, with conditions where no signaling related to cell cycle was detected between the top 10 functions, in contrast with HT-29, where these functions play an important role in the proteomic profile analyzed. 

### 2.3. Comprehensive Analysis of Cell Cycle and Apoptosis Proteomic Profiles

**Figure 5:**
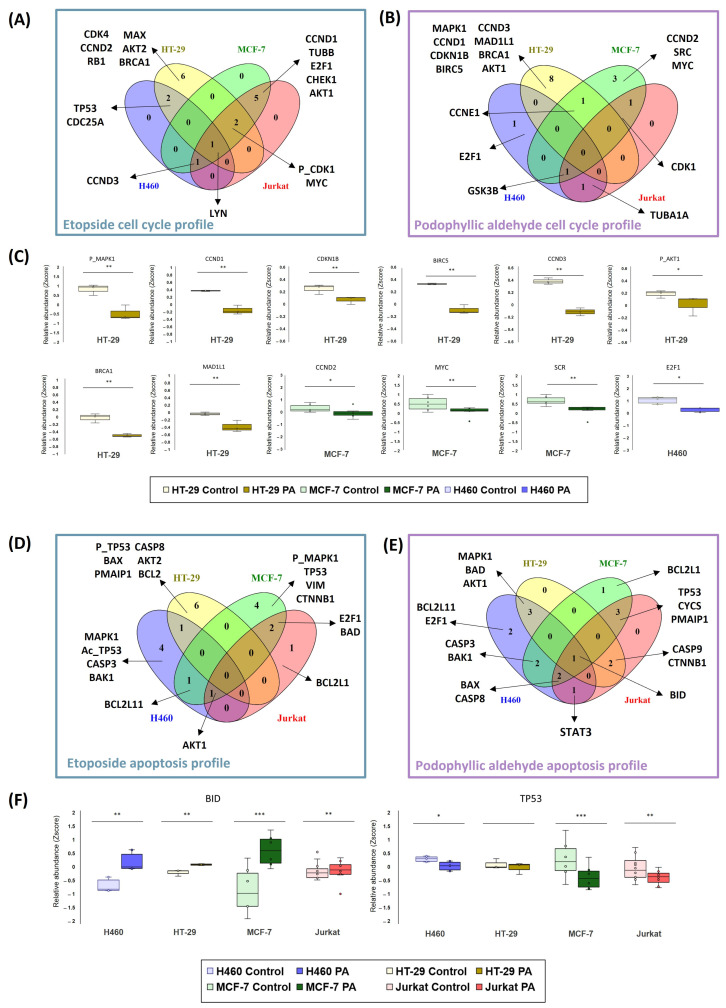
Determining etoposide and podophyllic aldehyde cell cycle and apoptosis profiles. Panels’ (**A**,**B**,**D**,**E**) Venn diagrams combine results from the four tumor cell lines studied based on cellular process (cell cycle and apoptosis). Panel (**C**) shows the levels of proteins detected for PA in cell cycle analysis of each cell line. Panel (**F**) shows the levels of proteins BID and TP53 detected for the four cell lines with PA. * = *p* < 0.05; ** = *p* < 0.01; *** *p* < 0.001.

Functional analysis approaches were also used to examine the differences and similarities between ETO and PA in their cell cycle and apoptosis profiles (Figure 5). 

These two processes were selected given the described mechanism of action of PA in previous works [15], the existing literature on ETO [9,10] and the findings of the global functional enrichment presented in the previous section (Figure 5). All the proteins analyzed from the microarray were functionally enriched, and pathways corresponding to the processes under study were selected (see Section 4.4.2 in Materials and Methods). The proteins that were significant for each cell line were compared with the proteins of each pathway, and the results were merged between all cell lines. The Venn diagrams in Figure 5 (panels A, B, D, and E) represent the unique features of each cell line, including H460 (blue), HT-29 (yellow), MCF-7 (green), and Jurkat (red), as well as the intermediate regions that match one, two, three, or all cell lines. 

In general, different proteins have been detected for each condition of compound tested and for each cell line in the cell cycle analysis (Figure 5A,B), highlighting the different mechanisms observed previously in this study. Our first insight was that we were able to observe that the HT-29 line is very different from the others in terms of its particular signaling. This fact was observed in both compounds (six and eight proteins, in particular, for ETO and PA, respectively). On the other hand, it appears that the H460 cell line is the least sensitive, as shown in the cell cycle profile. In this case, only three and four proteins were detected for PA and ETO, respectively, and concretely, in the ETO condition, none of the proteins belonged to a unique signaling for the compound in this cell line. This was repeated in the Jurkat cell line in signaling for PA and ETO. In contrast to the results for the H460 line, in the case of ETO in the Jurkat cell line, more proteins were detected for this cell line, but these overlapped with other cell lines’ signaling (nine proteins). In terms of these proteins in the Jurkat cell line, there was a total overlap with MCF-7 proteins: both cell lines shared this signaling. 

To further investigate PA signaling, Figure 5C represents the relative abundances (Z-score) of the specific proteins of each cell line concerning the control. Proteins for HT-29 were related to cyclin-type proteins responsible for cell cycle control (CCND1, CCND3, and CDKN1B), BRCA1 (e.g., as a modulator when DNA was damaged), signaling related to proliferative pathways (MPK1 and AKT1), and other proteins related to the mitotic spindle (BIRC5 and MAD1L1). When HT-29 protein levels were analyzed, a decrease in signaling was observed in the significative proteins for this pathway. A similar scenario was detected in proteins that were unique for MCF-7 (CCND2, MYC, and SRC) and H460 (E2F1). 

We also emphasized apoptosis signaling to decipher differences and similarities between the two compounds. In both cases, a heterogeneous signaling between compounds and cell lines was obtained. 

When inspecting the signaling for ETO (Figure 5D), it can be seen that all lines have different significant proteins. Compared with cell cycle results, HT-29 presented again the most particular signal, with six significative proteins. In this case, even the Jurkat cell line showed a particular deregulation (BCL2L1). In H460 and MCF-7, four significative proteins were detected. These two cell lines seem to share a profile related to MAPK and p53 pathways, as levels of different isoforms of the proteins were affected in these two cell lines (MAPK1 and acetyl-p53 in H460 and phospho-MAPK1 and p53 in MCF-7).

The results of the apoptosis profiles for PA in the four tumor cell lines studied were quite different (Figure 5E). Again, signaling appears to be shared between cell lines more than is the case for ETO. Only H460 and MCF-7 showed an apoptotic signal of their own, represented by only two and one protein, respectively. For this reason, in this scenario, we have focused the analysis on those proteins that appear in common between the tumor cell lines in order to observe the global tendency. Examples of common proteins are represented in Figure 5F. In the case of the BID protein, this BCL-related protein appears significantly upregulated in all the cell lines studied. Other proapoptotic proteins, such as BAD, BAX, and BAK1, were detected in the shared signaling of the cell lines. Also, caspases (CASP3, CASP8, and CASP9) were found in different cell lines. Another remarkable example found was TP53, which was also highlighted in ETO signaling. In this case, it could be observed that PA decreased the relative levels of TP53.

**Figure 6:**
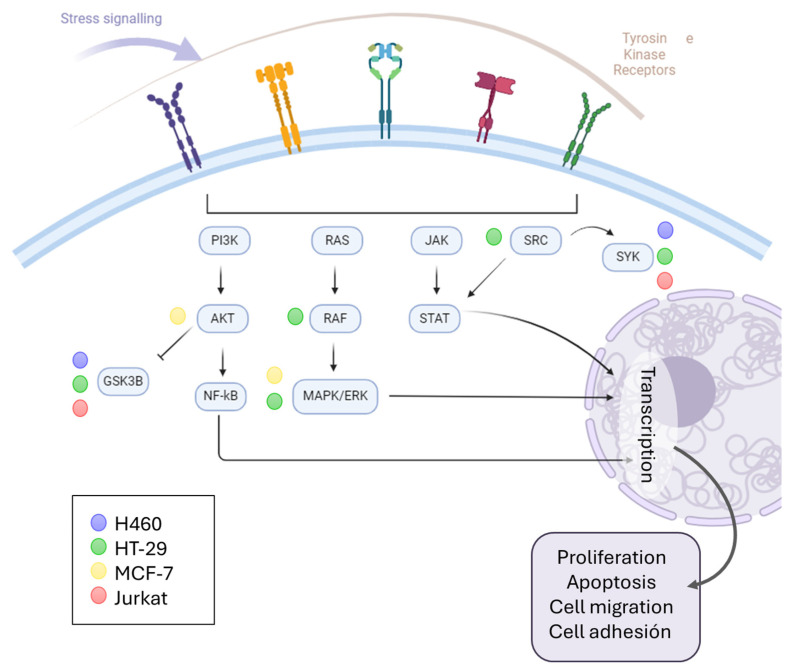
Schematic representation of kinase signaling, pointing out the proteins deregulated by PA.

Based on the results, it can be seen that the kinase signaling pathways were activated. For this reason, we wanted to find out more about the signaling mediated by PA and whether a relationship could be established between the cell type and the activity of this compound (Figure 6). For this purpose, functional enrichment was carried out through gene ontology on the proteins that were significantly deregulated when the cells were treated with PA, and those with a function in “protein kinase activity” (GO: 0004672) were selected. Many of the proteins matched the cyclin-dependent kinases (CDKs) discussed above. However, others corresponded to signaling downstream of membrane receptor tyrosine kinases. In this case, it appears that the MCF-7 cell line is the one most affected by PA treatment. Other lines (H460 and Jurkat) share signaling with MCF-7 through the GSK3B and SYK proteins, and the HT-29 line deregulated the AKT and MAPK proteins. In general, it can be observed that these signaling pathways that modulate processes of proliferation, apoptosis, migration, or cell adhesion, among others, are affected by PA.

## 3. Discussion

In this work, the antitumor scenario of the podophyllotoxin derivative named podophyllic aldehyde (PA), a compound that has demonstrated its cytotoxic potential, has been explored. Comparison with etoposide (ETO), a drug used in clinical practice that shares the same chemical structural basis, has provided interesting insights into the profile of this lead compound. Previous approaches to PA, in terms of the structure–activity relationship, have yielded some conclusions about its cytotoxic profile, but the intracellular signaling of this compound had not been explored so far [14,15]. Different solid or hematological tumors have also not been previously considered beyond cell viability values.

To date, the influence on cell viability and the antimitotic profile of PA has been studied among tumor cell lines of different natures, remarking on its selectivity and demonstrating the success of its further chemomodulation through different derivatives [16]. In this case, comparative study between different cell lines of the same tumor type made possible the observation of a certain selectivity between cell lines, as well as a tendency towards greater cytotoxicity in solid tumors versus hematological tumors. This relationship was inverted in the case of etoposide, which highlights the different mechanisms of action of the two molecules, as previously detailed in the literature [9]. When their influence on the PA cell cycle was previously studied, a total blockage in the G2/M phase has always been detected, as in the results obtained in this study with solid tumor cell lines. This cell cycle arrest was assigned to antimitotic activity by tubulin assembly assays [15]. Interestingly, despite being cytotoxic in the Jurkat cell line, PA did not show an antimitotic profile under the conditions studied. This new finding and differential profile between solid and hematological tumor cells will undoubtedly be taken into consideration for further studies.

Nevertheless, the proteomic profile has revealed novel insight into the cell cycle influence. Looking at the proteomics profile derived from the activity of PA and ETO, similarities and differences have been observed. The first analysis based on the proteomic profile provided by microarray (162 proteins studied) revealed that PA presented a prominent effect in cell cycle progress in the HT-29 cell line, according to results of a DNA content assay. However, cell lines H460 and MCF-7 obtained signaling for proliferation pathways (AKT and MAPK signaling) but not directly to mitotic assembly. For their part, cell cycle processes were represented in all ETO signaling for the cell cycle studies; this finding was in concordance with what was observed in the cell cycle analysis.

Investigating HT-29 more deeply, it was observed that cell cycle functions between the 10 most highlighted in enrichment appeared up to three times. This fact was subsequently reflected in the particular analysis of the specific proteins that constitute the signaling pathway. The HT-29 cell line treated with PA presented a very particular signaling that was different from the other lines. Cyclins and proteins specific to cell cycle control in the G2/M phase were among the proteins that were found to be significant. This differed from ETO signaling, which presented particular signaling in this cell line related to early stages of the cell cycle, such as CCND2 and CDK4. According to these data, it seems that PA in HT-29 presents a marked antimitotic effect and is very different from the effect of ETO. These results are consistent with the literature and previous findings about PA [15]. 

In contrast, when looking at the other two solid tumor lines studied, another proteomic pattern related to PA activity was observed. The signaling for H460 and MCF-7 in the cell cycle profile and the proteins observed in previous ETO profiles, such as CCND2 or E2F1, resulted in significant dysregulation. It has recently been shown that this signaling is more closely related to DNA topoisomerase II inhibition than to the antimitotic stage, suggesting another profile for PA in H460 and MCF-7 in terms of cell cycle influence. In terms of the Jurkat cells, this was the cell line where both compounds shared the highest number of deregulated proteins. However, PA did not retrieve independent signaling by itself. In this case, the particular proteomic analysis of the cell cycle also did not yield conclusive results about the cell cycle activity. If we compare its results with those of the cell cycle assays, it seems that this compound, in this cell line, does not affect either mitotic machinery nor the functions related to other phases of the cycle. Certainly, together with the cytotoxic activity shown, this is one of the perspectives to be studied in future assays performed with this cytotoxic compound.

The analysis of the apoptotic signal seems to be more heterogeneous between the lines and between the two compounds. It appears that in all cases, there is activation of caspases and other apoptotic modulators of the BCL2 family. In this case, it is not possible to correlate the overall profile with the particle profile as well as we have been able to observe in the cell cycle. However, given the high number of apoptosis-related proteins deregulated by PA, we have been able to determine that, independently of the underlying cell cycle mechanism, an apoptosis-mediated cell death signal is triggered in the cell lines tested. The proteomic profile of the natural product podophyllotoxin was previously studied, and the results are consistent with some of the findings related to PA in our research [30,31,32]. However, the duality of the cyclolignan skeleton or the possibility of triggering other activities or mechanisms of action by cyclolignans remains a concern that we also try to shed light on in our study. 

One of the findings of this study is the prominent role that kinases play in the signaling mediated by these compounds. This signaling is tightly connected to apoptosis. As we observed in our study, it seems that all the cell lines trigger this kind of programmed cell death. In addition to the active demonstration that ETO is a DNA topoisomerase II inhibitor [10], this structural modification does not always drive the change in activity with respect to podophyllotoxin. Some authors have found an antimitotic effect of cyclolignan derivates with etoposide-like substitution [33,34,35]. In contrast, and similar to ETO, numerous derivates containing C-7β podophyllotoxin derivates have demonstrated their DNA topoisomerase II inhibition profile [36]. Therefore, it is not surprising that a structural modification such as the one in PA—that is, the absence of the lactone present in the natural product—can give rise to different mechanisms of action depending on the cell type, as occurs in this present research.

Concerning the programmed cell death triggered by ETO and PA, our study was focused on apoptosis, the most preserved, induced cell death mechanism associated with antitumoral drugs [37] (in general) and with podophyllotoxin-related compounds [38,39,40,41]. In our case, we demonstrated apoptosis induction independently of cell cycle effect, cytotoxic profile, or tumoral cell line. In our results, looking at the overall profile of the compounds, we have observed many functions associated with many others related to immune processes. Specifically, in H460 and MCF-7, these PA conditions were tested where no association of this compound with the cell cycle was observed. Similar testing was carried out in the case of solid tumor cells in ETO. Given these findings and taking into account that other cytotoxic compounds derived from natural sources have been able to induce this immunogenic death process [42], the role of podophyllotoxin and its derivatives as positive immunomodulators in response to the cell death process can be considered for future research on podophyllotoxin and its derivatives. In this sense, in future assays and screenings of this type of compounds, it would be necessary to include tests that help us to understand, more specifically, the intracellular activity related to cellular stress and the release of cellular patterns associated with damage.

As a result of our study, it is possible to verify, in a fast and efficient way, the different profiles of two compounds that are very close in their structural relationship and mechanism of action, since they are two antitumor compounds with an influence on the cell cycle. This highlights the versatility and robustness of targeted proteomics for drug discovery [43,44]. Similarly, other high-throughput screening techniques can be proposed for drug analysis and correlation of data at the level of other omics (genomics, metabolomics, etc.), which have been shown to play a fundamental and complementary role to proteomics in elucidating drug action mechanisms [45,46]. Therefore, future studies will be aimed at monitoring the parameters that may provide more information on the antitumor profile.

## 4. Materials and Methods

### 4.1. Chemical and Biological Materials

Podophyllotoxin, as starting material, was obtained from resin of rhizome of *Podophyllum peltatum* as previous described [16]. Further transformation into podophyllic aldehyde was conducted following the synthetic procedure described previously by us [15]. Etoposide was purchased from Sigma-Aldrich (#E1383, Saint Louis, MO, USA). Compounds were dissolved in DMSO (10^−2^ M) and further dilutions were performed in PBS Na^+^/K^+^. Final concentration of DMSO in assays were <0.001%. 

Dimethyl sulfoxide (DMSO) was obtained from Merck (Darmstadt, Germany). Bovine serum albumin (BSA), trypan blue, urea, and 3-(4,5-dimethylthiazol-2-yl)-2,5-diphenyltetrazolium bromide (MTT) were purchased from Sigma-Aldrich (St. Louis, MO, USA), and 96-well plates and BCA protein assay kit were purchased from Thermo Scientific (Rockford, IL, USA). Heat inactivated fetal bovine serum (FBS), L-glutamine, penicillin–streptomycin (P-S), and 0.25% trypsin-EDTA were purchased from Gibco^®^ (Gran Island, NY, USA), and 6-well, clear, flat-bottom plates were purchased from Corning (Corning, NY, USA). Cycloscope™ reagent for cell cycle analysis was purchased from Cytognos (Salamanca, Spain).

### 4.2. Tumor Cell Lines Culture

All the human cell lines [H460 (ATCC^®^ HTB-177, Manassas, VA, USA); A549 (ATCC^®^ CCL-185); Caco-2 (ATCC^®^ HTB-37™); HT-29 (ATCC^®^ HTB-38™); MCF-7(ATCC^®^ HTB-22); MDA-MB-231 (ATCC^®^ HTB-26); Jurkat, T-cell leukemia (DSMZ ACC 282, Braunschweig, Germany) and Ramos B-cell lymphoma (ATCC^®^ CRL-1596™)] were cultured at 37 °C in a humidified CO_2_ incubator (5% CO_2_) in complete RPMI media (hematological cell lines Jurkat and Ramos) or DMEM media (adherent cells H460, A549, Caco-2, HT-29, MCF-7, and MDA-MB-231) (both supplemented with 10% (*v*/*v*) FBS and 1% (*v*/*v*) penicilin–streptomycin). When the cells reached 80% confluence, they were subcultured. For this purpose, adherent cells were washed with PBS and treated with 1 mL 0.25% trypsin-EDTA to detach them. Once collected, cells were centrifugated at 1200 rpm for 5 min. Suspension cells were collected directly from the cell culture plate and centrifugated in the conditions mentioned above. When necessary, the cells were counted using a Neubauer counting chamber and dyed with Trypan Blue. 

### 4.3. MTT Assays for Cytotoxicity Determination and Cell Viability

Using previous protocols reported by us [16], etoposide and podophyllic aldehyde viability assays were carried out for the cell lines detailed above. For each cell line, a different number of cells were cultured in 96-well plates before the corresponding drug was added: H460, A549, and MDA-MB-231 1000 cells; HT-29, Caco-2, and MCF-7 2500 cells; Jurkat and Ramos 10,000 cells. Adherent cells were seeded 24 h before the assay to allow attachment in 100 µL media. Cells were incubated with the corresponding stimuli (ETO and PA) for 72 h at different dilutions (100 µM of media containing 1 μM–0.01 μM of the drugs). Each condition was assayed in triplicate, performing the experiment twice. Once these periods of time elapsed, the supernatant was replaced with fresh medium (100 µL) and 20 μL per well MTT (5 mg/mL) was added in darkness. After 4 h MTT incubation, the supernatant was removed and 200 μL DMSO per well was added to dissolve formazan. Absorbance at 570 nm was determined by Gen5™ software (https://www.agilent.com/en/product/microplate-instrumentation/microplate-instrumentation-control-analysis-software/imager-reader-control-analysis-software/biotek-gen5-software-for-detection-1623227) (BioTek U.S., Winooski, VT, USA). The viability (percentage) for each concentration was calculated by the following Equation (1):

(1) $$\frac{Abs.\;of\;cells\;with\;drug-Abs.\;of\;cells\;without\;drug}{Abs.\;of\;growth\;medium\;with\;drug-Abs.\;of\;growth\;medium\;without\;drug}\times 100$$

Equation (1): Calculation of cell viability in MTT assays.

### 4.4. Cell Cycle Evaluation by Flow Cytometry

DNA content by flow cytometry was conducted following the protocol optimized by us [16] with slight differences. A total of 1 × 10^5^ cells per well were seeded in 60 mm cell culture dishes with 5 mL of media. Cells were exposed to the drugs (1 µM) after 24 h of incubation. Then, the cells were collected (Jurkat) or detached using 500 µL of Tripsin (H460, HT-29, and MCF-7) and centrifuged three times at 1200 rpm for 5 min. With each centrifugation, the supernatant was discarded and replaced with PBS (5 mL). In the final centrifugation, PBS was replaced with 200 µL of Cycloscope™ Reagent, and samples were incubated in darkness for 15 min. The results were obtained by using a flow cytometer BD Accuri™ C6 acquiring 100,000 events per condition (performed in duplicate) and analyzed with Infinicyt^TM^ (Cytognos, Salamanca, Spain) determining the % of cells in each cell cycle phase.

#### 4.4.1. Cell Lysis, Labelling, and Protein Array Processing

Cell line pellets (H460, HT-29, MCF-7, and Jurkat) treated with ETO and PA (1 µM) for 24 h were collected and stored at −80 °C before lysis. For lysis, a total protein lysis buffer together with phosphatase and protease inhibitors was used [29]. After adding lysis buffer, the pellets were sonicated, with three pulses of 10 s with an incubation interval on ice of 1 min between pulses for each pellet. Finally, the pellets were centrifuged at 12,000× *g* for 15 min at 4 °C to obtain the supernatant containing the protein lysate. The BCA protein assay kit was used for protein quantification.

Hereafter, protein lysates were labeled with biotin and stored at −4 °C until incubation in antibody arrays. Incubation in antibody arrays was performed following previous protocols adapted by us [47]. Microarrays blocking, sample preparation, and sample incubation was performed as described in [47]. 

#### 4.4.2. Image Acquisition and Raw Data Processing

The arrays were scanned with SensoSpot Fluorescence (Miltenyi Imaging GmbH, Radolfzell, Germany). The generated TIFF images were analyzed using GenePix Pro 6.0 software (Molecular Devices, San Jose, CA, USA). Parameters were set to quantify the intensity values of the Cy3 fluorochrome (λ = 532 nm). The fluorescence signal of the protein microarrays was corrected by subtracting the background signal and then transformed to Z-score. Proteins and their codification are collected in Supplementary File S2 and antibody references used for microarray in [29]. Each protein analyte was evaluated in two biological replicates each with three technical replicates. Based on previous reports [47], negative fluorescence signal was transformed to zero. As expected, the signal distribution was not normal (Supplementary File S3). Therefore, nonparametric Mann–Whitney test was used to compare the protein relative abundance (Z-score) between groups (experiment ETO or PA versus control). Functional enrichment analysis was conducted with significative proteins using Reactome enrichment. Resulting from functional enrichment, Reactome pathway terms were selected for signaling pathways involved in apoptosis (HSA-109581) and cell cycle (HSA-1640170) for further analysis. 

### 4.5. Determination of Protein Profiles 

The determination of differential protein profiles is performed by assessing the relative abundance of intracellular proteins by antibody-based protein arrays. The protein arrays used in this work were designed following previous reports [24,29] and functionalized and printed at the Functional Proteomics Service of the Cancer Research Center according to the procedures for the preparation of protein arrays as described previously [29,30,31]. 

## 5. Conclusions

The compound podophyllic aldehyde was analyzed for its potential as an antitumoral agent using cell viability, cell cycle influence, and targeted antibody microarray techniques, and it was compared with the approved drug etoposide. The results showed that it is a promising compound derived from podophyllotoxin, presenting a potent and selective cytotoxicity and showing great success in cell cycle arrest. One of the key findings of this study is that the tumor activity of this compound varies depending on the type of tumor studied, which is attributed to its different effects on the cell cycle. Among the cell lines, HT-29 appears to be the most sensitive, and cellular signaling reflects an antimitotic effect in this cell line, following the mechanism of action of the natural product podophyllotoxin. In contrast, other cell lines (MCF-7 and H460) did not present this profile, being more similar to the ETO profile and revealing other interesting antitumor features such as kinase activity or the activation of immune mechanisms. Hence, by comparing PA with the ETO drug, it has been possible to understand the structure–activity relationship of the cyclolignan derivatives and how the structural characteristics may influence the final activity of the different tumor lines. Overall, our study has demonstrated the importance of chemomodulation of natural compounds as antitumor agents, as well as the relevance of a systematic proteomic screening to unveil the mechanisms of action in an efficient and insightful manner. 

## Figures and Tables

**Table 1:** Cytotoxicity values (IC_50_, µM) ± standard deviation (SD) of etoposide (ETO) and podophyllic aldehyde (PA).

	Lung Cancer		Colorectal Cancer		Breast Cancer		Hematologic Neoplasia	
	A549	H460	HT-29	Caco-2	MCF-7	MDA-MB-231	Jurkat	Ramos
**ETO**	0.802 ± 0.231	0.842 ± 0.39	1.25 ± 0.02	1.06 ± 0.64	0.751 ± 0.002	0.256 ± 0.046	7.09 ± 0.19	6.45 ± 0.53
**PA**	0.0371 ± 0.0134	0.088 ± 0.011	0.0283 ± 0.0015	0.197 ± 0.058	0.0663 ± 0.0182	0.430 ± 0.023	5.23 ± 0.17	>100

## Data Availability

The original contributions presented in the study are included in the article/Supplementary Materials, further inquiries can be directed to the corresponding author/s.

## Supplementary Materials

The supporting information can be downloaded at: https://www.mdpi.com/article/10.3390/ijms25094631/s1.
