# Supplementary material for: Podophyllic Aldehyde, a Podophyllotoxin Derivate, Elicits Different Cell Cycle Profiles Depending on the Tumor Cell Line: A Systematic Proteomic Analysis
Source: Int J Mol Sci. 2024 Apr 24;25(9):4631. doi: 10.3390/ijms25094631 (PMC11083757; doi:10.3390/ijms25094631)
Supplement: Supplementary file 1 [file ijms-25-04631-s001.zip › Hernández2024_IJMS_FileS3.pdf]

Suplemmentary File S3

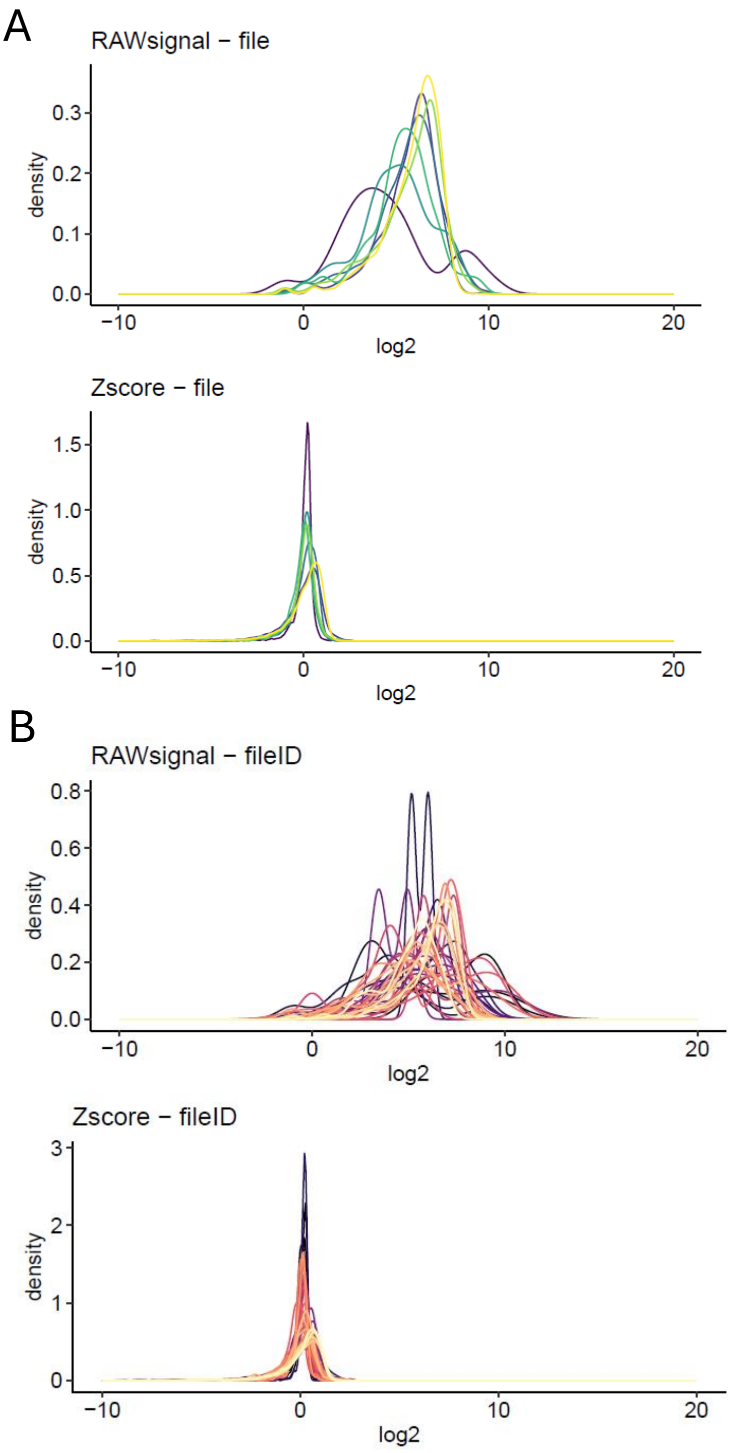

Density Plots for signal distribution of microarrays (A) and subarrays (B)

Suplemmentary File S3

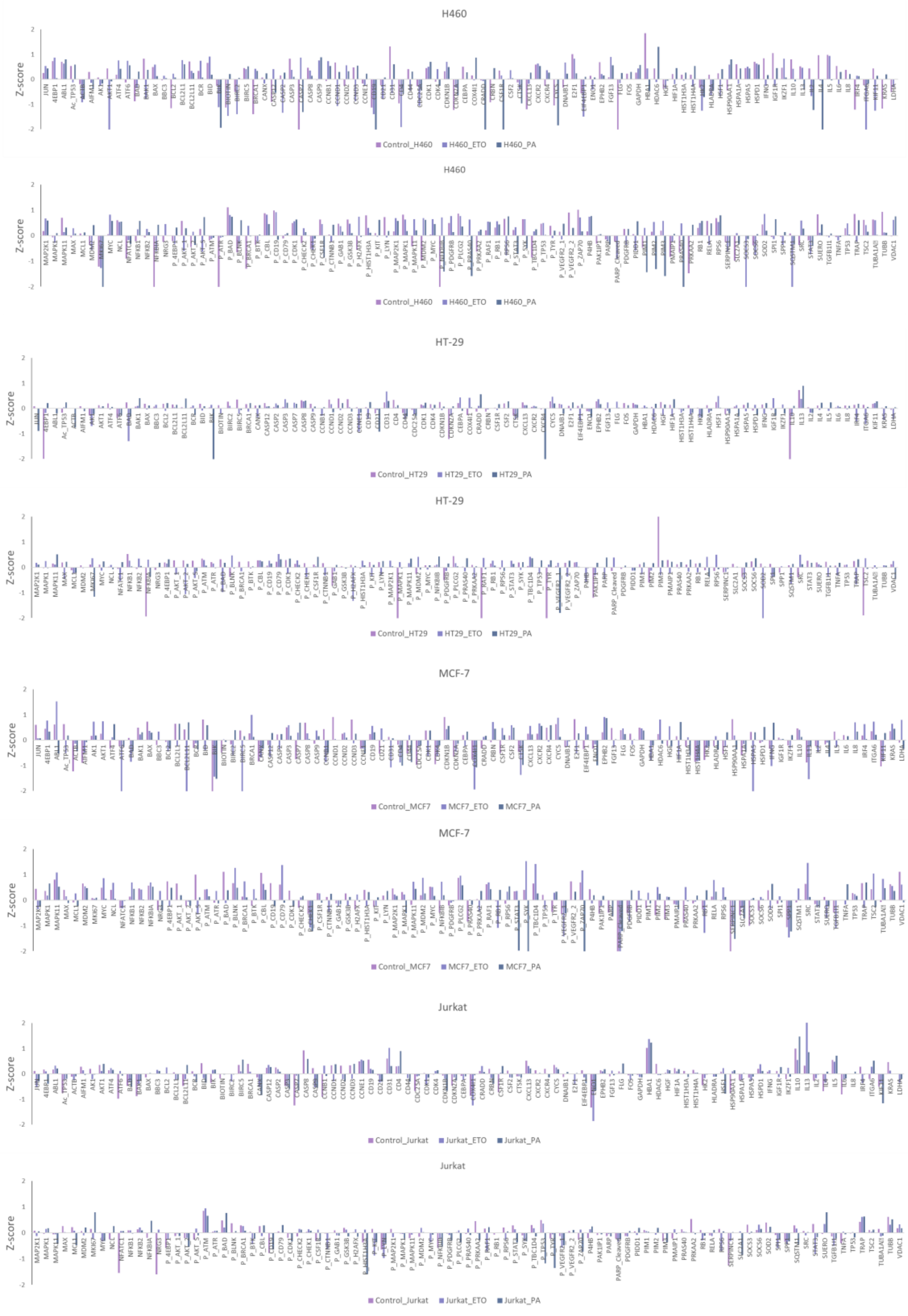

Relative abandance distribution (Zscore) for proteins in control conditions and drugs: etoposide (ETO) and podophyllic aldehyde (PA) in the for tumoral cell lines tested in this work (H460, HT-29, MCF-7 and Jurkat)
